# Association between NPAR and cognitive impairment in peritoneal dialysis patients

**DOI:** 10.3389/fnut.2026.1752923

**Published:** 2026-03-04

**Authors:** Conghui Liu, Feng Shao, Xiaoqi Wang, Jiajie Cai, Zhongxin Li

**Affiliations:** Department of Nephrology, Beijing Luhe Hospital, Capital Medical University, Beijing, China

**Keywords:** chronic kidney disease, cognitive impairment, NPAR, peritoneal dialysis, risk factors

## Abstract

**Background:**

The neutrophil percentage-to-albumin ratio (NPAR) is a novel inflammatory marker. This study explores its association with cognitive impairment (CI) in peritoneal dialysis (PD) patients.

**Methods:**

In this cross-sectional study, 152 PD patients were categorized into CI (Montreal Cognitive Assessment (MoCA) score <26) or non-CI (MoCA ≥26) groups.

**Results:**

CI was present in 66.45% of PD patients. Patients in the CI group had older age (63.01 ± 10.88 vs. 49.75 ± 12.74 years, *p* < 0.001), a high proportion of female individuals (43.56% vs. 23.53%, *p* = 0.016), and a higher NPAR (1.94 ± 0.24 vs. 1.80 ± 0.24, *p* = 0.001). In addition, patients in the CI group had lower levels of education (8.24 ± 2.97 vs. 11.55 ± 3.45 years, *p* < 0.001), serum albumin (36.29 ± 3.56 vs. 37.75 ± 2.60 g/L, *p* = 0.010), potassium (4.30 ± 0.71 vs. 4.51 ± 0.53 mmol/L, *p* = 0.039), creatinine (865.79 ± 274.38 vs. 1099.92 ± 293.86 umol/L, *p* < 0.001), and phosphorus (1.43 ± 0.41 vs. 1.68 ± 0.44 mmol/L, *p* = 0.001). Multivariate logistic regression analysis revealed that NPAR, age, serum phosphorus levels, and education were significant independent determinants of CI. The area under the curve (AUC) for NPAR in predicting CI was 0.657, with a sensitivity of 0.496 and a specificity of 0.745 (*p* = 0.002). When age, NPAR, blood phosphorus levels, and education were combined, the AUC increased to 0.861, with a sensitivity of 0.822 and specificity of 0.745 (*p* < 0.001).

**Conclusion:**

CI in PD patients was found to be independently associated with elevated NPAR. The NPAR may serve as a potential biological indicator for identifying prevalent cases of CI, providing a basis for further exploration of early intervention strategies for CI.

## 1 Introduction

Cognitive impairment (CI) occurs due to dysfunction in one or more key cognitive domains such as memory and attention, learning ability, and executive function. Individuals with chronic kidney disease (CKD) are at a markedly increased risk of developing CI, a risk that intensifies as renal function declines, particularly evident among dialysis patients (1, 2). Despite the advantages of peritoneal dialysis (PD), including reduced hemodynamic instability and lower anticoagulation requirements compared to hemodialysis, previous studies have shown that cognitive impairment remains prevalent among PD patients. Meta-analytic findings revealed that 47.7% (95% CI: 35.8–59.9%) of PD patients exhibit CI (3). CI in PD patients is associated with a higher incidence of peritonitis, increased hospital admissions, and an elevated all-cause mortality rate, ultimately compromising their quality of life and clinical outcomes (4). The assessment of cognitive function is complex; therefore, identifying new biomarkers for CI is particularly important.

The neutrophil percentage-to-albumin ratio (NPAR) is a newly recognized biomarker associated with systemic inflammation and immune status (5). Compared to other inflammatory ratios, such as the neutrophil-to-lymphocyte ratio (NLR) and C-reactive protein-to-albumin ratio (CAR), the NPAR combines the neutrophil percentage (which provides a more stable reflection of systemic inflammatory levels than the absolute neutrophil count, which can change rapidly due to infection or stress) with serum albumin (ALB, a key mediator in assessing both nutritional status and anti-inflammatory responses). In previous studies, the NPAR has been extensively used to evaluate disease risk and predict clinical outcomes (6–10). However, only a few studies have focused on PD patients. Gao et al. (11) demonstrated that the NPAR serves as an independent risk indicator for overall and cardiovascular mortality in PD patients. In the field of cognitive function, a recent study found that the NPAR is associated with CI in elderly people aged 60 and above (12). Notably, the unique pathophysiology of PD (e.g., chronic low-grade inflammation and malnutrition-inflammation complex syndrome) may render the NPAR more relevant to CI than the NLR or CAR, as it captures both persistent inflammatory dysregulation and impaired nutritional-anti-inflammatory defense. However, the relationship between the NPAR and CI has not yet been clarified in patients undergoing PD. In summary, our study is the first to explore the association between the NPAR and cognitive impairment in individuals undergoing PD and its predictive value for the occurrence of CI, further providing evidence for the diagnosis and intervention of CI.

## 2 Materials and methods

### 2.1 Study design and participants

**Figure 1:**
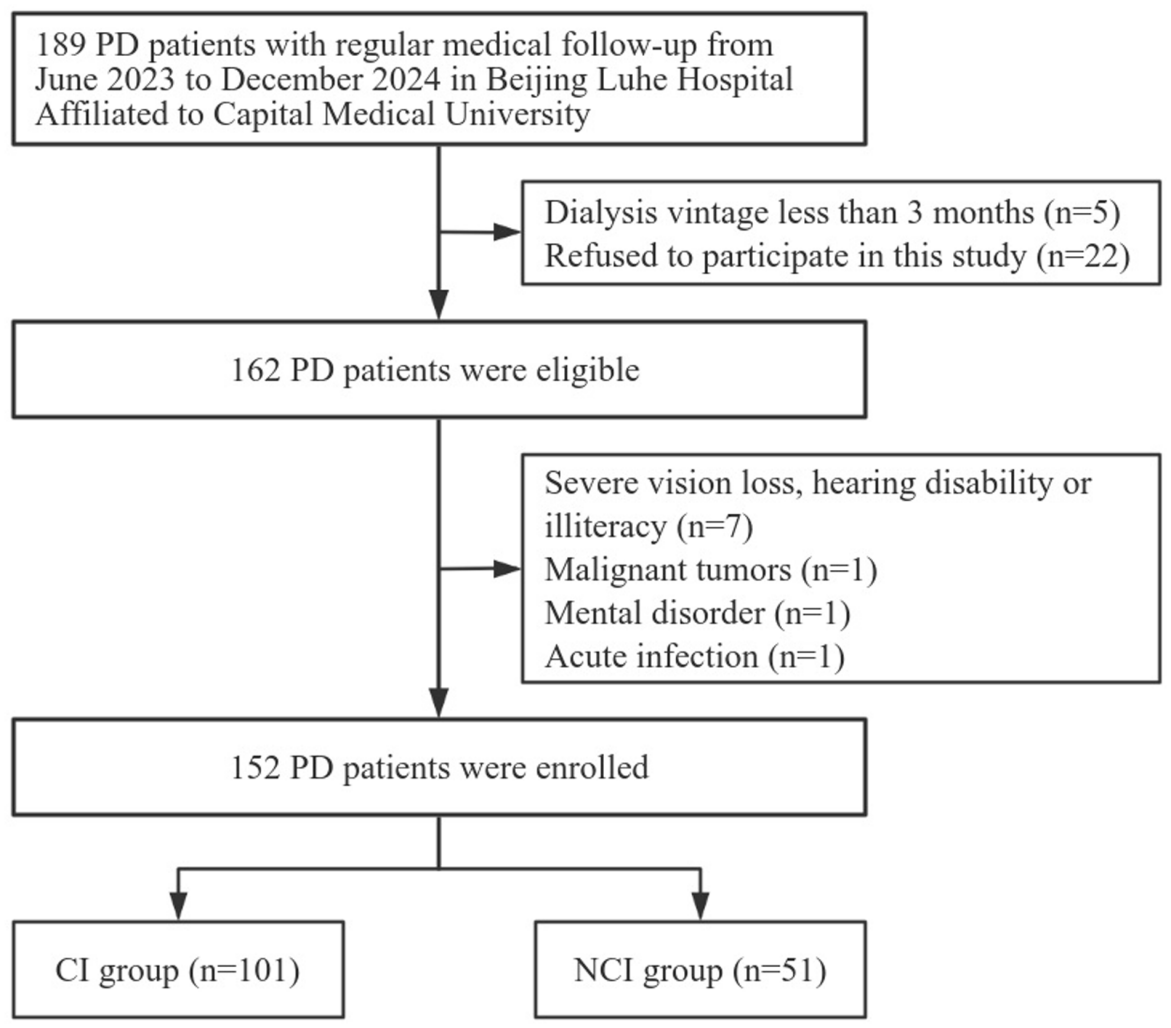
Flowchart of patient recruitment and grouping. PD, Peritoneal dialysis; CI, Cognitive impairment; NCI, Non-cognitive impairment.

This study used a cross-sectional observational design, focusing on individuals undergoing PD treatment. From June 2023 to December 2024, all patients with end-stage renal disease who underwent PD at Beijing Luhe Hospital, affiliated with Capital Medical University, were enrolled. The inclusion criteria were as follows: (1) age ≥ 18 years, (2) have been on PD for at least 3 months, and (3) have agreed to participate in this study. The exclusion criteria were as follows: (1) acute infection, autoimmune disease, inflammatory bowel disease, cancer, or serious organ dysfunction potentially affecting inflammatory biomarkers. (2) Impairments such as profound vision loss, communication difficulties, inability to read, mental illnesses, or upper-extremity disabilities that impede study involvement. (3) Patients who had been assessed using the same cognitive scale in the last 3 months. Among the 189 screened PD patients, 152 were selected for participation in the study. Figure 1 depicts the flowchart outlining the recruitment of participants for the study.

This study was approved by the Ethics Committee of Beijing Luhe Hospital (2023-LHKY-012-02). Informed consent was obtained from all participants. All methods were carried out in accordance with the Declaration of Helsinki.

### 2.2 Clinical and laboratory characteristics

After admission, the patients’ demographic data and comorbid conditions were documented. This included sex, age, dialysis duration, underlying renal disease, hypertension status, diabetes mellitus history, cardiovascular disease (CVD) history, smoking history, and years of education. If any of the following conditions were present, they were recorded as CVD: angina, congestive heart failure classified as NYHA III–IV, transient ischemic attacks, prior myocardial infarction or stroke, and peripheral arterial disease.

Anthropometric data were also recorded. Laboratory tests performed at baseline included leukocyte subsets (neutrophils, lymphocytes), platelets, C-reactive protein (CRP), serum albumin, lipid parameters, serum electrolytes (potassium, sodium), total carbon dioxide (tCO₂), renal function indicators (creatinine, urea), corrected calcium, phosphorus, intact parathyroid hormone (iPTH), ferritin, and total weekly Kt/V.

### 2.3 NPAR calculation

The NPAR was calculated by dividing the neutrophil percentage of leukocytes by the albumin concentration [neutrophil percentage (%)/albumin (g/L)].

The NLR was computed by dividing the neutrophil count by the lymphocyte count, and the CAR was calculated by dividing the CRP concentration by the albumin concentration. The units were expressed as ×10^9^/L for cell counts, mg/L for CRP, and g/L for albumin.

### 2.4 Measurement of CI

Cognitive function was assessed in a quiet and distraction-free environment by a trained evaluator using the Montreal Cognitive Assessment (MoCA) (Beijing version). This tool evaluates seven key cognitive domains, namely language skills, delayed recall, abstract thinking, naming, attention, visuospatial/executive functioning, and orientation. To minimize the impact of limited education, a score increment of one was applied for those with ≤12 years of schooling. The MoCA scores ranged from 0 to 30, with higher scores reflecting better cognitive performance. Cognitive impairment (CI) was characterized by a MoCA score less than 26 (13).

### 2.5 Statistical analysis

All statistical analyses were performed using the IBM Statistical Package for Social Sciences (SPSS, version 26.0; IBM, NY, USA). For normally distributed variables, data are shown as mean ± standard deviation. For non-normally distributed variables, medians and interquartile ranges (IQR) are used. Parametric continuous variables were assessed using Student’s *t*-test, and the Mann–Whitney U-test was used for non-parametric variables. The chi-square (*χ*^2^) test was used to analyze categorical variables. To explore factors independently associated with CI, a forward likelihood ratio method was used to develop the binary logistic regression model. The performance of significant predictors was assessed using the receiver operating characteristic (ROC) curve analysis. A combined predictive model for CI was constructed based on the logistic regression coefficients of the four independent determinants (age, education, NPAR, and serum phosphorus) identified in the multivariate logistic regression analysis. A combined risk score was calculated for each patient using these coefficients, and the ROC curve for the combined model was generated accordingly. Decision curve analysis (DCA) was performed to evaluate the clinical utility of predictive models. Statistical significance was defined as a two-tailed *p*-value of less than 0.05.

## 3 Results

### 3.1 Baseline characteristics

A total of 152 PD patients were included in the study. The mean age of the selected PD patients was 58.58 ± 13.11 years. Among the participants, 36.84% were women; the median PD duration was 28.00 (13.00, 57.75) months. Of the patients, 149 (98.03%) patients had hypertension, 90 (59.21%) had diabetes mellitus, and 85 (55.92%) had a history of CVD. Diabetes mellitus (*n* = 78), chronic glomerulonephritis (*n* = 44), and hypertension (*n* = 23) were the most common renal disorders. The mean MoCA score was 22.23 ± 5.70. Finally, CI was present in 66.45% of PD patients.

### 3.2 Comparison of clinical profiles and laboratory indicators between PD patients with and without cognitive impairment

All PD patients were categorized into two groups based on their MoCA scores: those with cognitive impairment (CI group, MoCA < 26) and those without cognitive impairment (NCI group, MoCA ≥ 26). Patients in the CI group were older than those in the NCI group (63.01 ± 10.88 vs. 49.75 ± 12.74 years, *p* < 0.001). The CI group also had a higher proportion of women (43.56% vs. 23.53%, *p* = 0.016). The CI group had a higher NPAR than the NCI group (1.94 ± 0.24 vs. 1.80 ± 0.24, *p* = 0.001). In addition, patients in the CI group had lower levels of education (8.24 ± 2.97 vs. 11.55 ± 3.45 years, *p* < 0.001), serum albumin (36.29 ± 3.56 vs. 37.75 ± 2.60 g/L, *p* = 0.010), potassium (4.30 ± 0.71 vs. 4.51 ± 0.53 mmol/L, *p* = 0.039), creatinine (865.79 ± 274.38 vs. 1099.92 ± 293.86 umol/L, *p* < 0.001), and phosphorus (1.43 ± 0.41 vs. 1.68 ± 0.44 mmol/L, *p* = 0.001). There were no differences in dialysis duration, body mass index (BMI), systolic blood pressure (SBP), diastolic blood pressure (DBP), hemoglobin levels, leukocytes, CRP, NLR, CAR, triglycerides (TGs), total cholesterol (TC), high-density lipoprotein cholesterol (HDL-C), low-density lipoprotein cholesterol (LDL-C), serum sodium, total carbon dioxide, serum urea, serum uric acid, serum corrected calcium, serum ferritin, and total Kt/V per week (Table 1).

**Table 1:** Comparison of clinical parameters between PD patients with different cognitive functional states.

Variables	CI group (*n* = 101)	NCI group (*n* = 51)	F/Z/χ^2^	*p*-value
Age (years)	63.01 ± 10.88	49.75 ± 12.74	1.523	<0.001
Female (*n*, %)	44 (43.56%)	12 (23.53%)	5.846	0.016
Dialysis vintage (months)	28.00 (12.50, 56.50)	28.00 (17.00, 64.00)	−0.459	0.647
Diabetes mellitus (*n*, %)	65 (64.36%)	25 (49.02%)	3.300	0.069
Hypertension (*n*, %)	100 (99.00%)	49 (96.08%)	0.371	0.542
CVD history (*n*, %)	66 (65.34%)	19 (37.25%)	10.849	0.001
Smoking history (*n*, %)	48 (47.52%)	27 (52.94%)	0.398	0.528
Education (years)	8.24 ± 2.97	11.55 ± 3.45	4.604	<0.001
BMI (kg/m^2^)	24.15 ± 3.80	25.20 ± 4.53	2.581	0.133
SBP (mmHg)	130.26 ± 20.46	131.84 ± 14.98	8.491	0.624
DBP (mmHg)	78.85 ± 11.77	82.61 ± 11.18	0.087	0.061
Hemoglobin(g/L)	115.92 ± 12.23	113.98 ± 10.52	0.686	0.335
Leukocytes (×10^9^/L)	7.58 ± 2.24	7.50 ± 2.21	0.014	0.819
Neutrophils (×10^9^/L)	5.36 ± 1.78	5.09 ± 1.73	0.031	0.366
Lymphocytes (×10^9^/L)	1.48 ± 0.62	1.61 ± 0.61	0.160	0.231
Platelets (×10^9^/L)	217.03 ± 64.74	219.16 ± 63.74	0.109	0.848
Serum CRP (mg/L)	3.31 (0.98, 8.95)	2.21 (0.79, 7.41)	−0.763	0.446
Serum albumin (g/L)	36.29 ± 3.56	37.75 ± 2.60	5.899	0.010
NLR	4.09 ± 1.89	3.51 ± 1.60	1.910	0.062
NPAR	1.94 ± 0.24	1.80 ± 0.24	0.105	0.001
CAR	0.10 (0.03, 0.25)	0.06 (0.02, 0.19)	−0.888	0.375
Serum TC (mmol/L)	4.19 ± 1.06	4.08 ± 0.84	1.499	0.505
Serum TG (mmol/L)	1.63 (1.04, 2.22)	1.39 (1.03, 2.11)	−1.216	0.224
Serum HDL-C (mmol/L)	1.03 ± 0.25	1.04 ± 0.31	1.338	0.945
Serum LDL-C (mmol/L)	2.53 ± 0.82	2.36 ± 0.56	4.103	0.175
Serum potassium (mmol/L)	4.30 ± 0.71	4.51 ± 0.53	2.250	0.039
Serum sodium (mmol/L)	138.54 ± 3.03	139.08 ± 3.03	0.280	0.307
Serum tCO2(mmol/L)	25.44 ± 2.28	25.34 ± 1.56	4.972	0.784
Serum creatinine (umol/L)	865.79 ± 274.38	1099.92 ± 293.86	1.815	<0.001
Serum urea (mmol/L)	21.88 ± 6.49	22.61 ± 6.19	0.049	0.506
Serum uric acid (umol/L)	341.91 ± 69.23	333.39 ± 68.37	0.590	0.473
Serum corrected calcium (mmol/L)	2.37 ± 0.21	2.34 ± 0.16	0.884	0.287
Serum phosphorus (mmol/L)	1.43 ± 0.41	1.68 ± 0.44	0.834	0.001
Serum ferritin (ng/mL)	195.35 (97.70, 342.25)	203.1 (82.50, 336.10)	−0.061	0.951
Serum iPTH (pg/dL)	152.30 (46.44, 262.58)	169.90 (89.60, 270.80)	−1.176	0.239
Total Kt/V per week	1.83 ± 0.33	1.80 ± 0.44	1.243	0.808

PD, peritoneal dialysis; CI, cognitive impairment; NCI, non-cognitive impairment; CVD, cardiovascular disease; BMI, body mass index; SBP, systolic blood pressure; DBP, diastolic blood pressure; CRP, C-reactive protein; NLR, neutrophil-to-lymphocyte ratio; NPAR, neutrophil percentage-to-albumin ratio; CAR, C-reactive protein-to-albumin ratio; TC, total cholesterol; TG, triglycerides; HDL-C, high-density lipoprotein cholesterol; LDL-C, low-density lipoprotein cholesterol; tCO2, total carbon dioxide; iPTH, intact parathyroid hormone.

### 3.3 Independent determining factors for CI according to multivariate logistic regression analysis in PD patients

To control for potential confounding variables affecting CI, a multivariate logistic regression analysis was conducted. This analysis aimed to identify independent factors contributing to CI in patients undergoing PD. Variables (such as age, sex, CVD history, education, serum ALB, NPAR, serum potassium, serum phosphorus, serum creatinine, *p* < 0.05) showed significant associations with cognitive impairment in the univariate analysis, and several previously reported variables (diabetes mellitus, dialysis vintage, DBP, and NLR) were included in the analysis as candidate variables. In the analysis, age, education, NPAR, and serum phosphorus levels were independently associated with CI in PD patients (Table 2).

**Table 2:** Independent determining factors for cognitive impairment according to the multiple logistic regression analysis in PD patients.

Variable	Univariate logistic regression		Multivariate logistic regression	
	*B* (95% CI)	*p*-value	*B* (95% CI)	*p*-value
Age (years)	1.100 (1.062–1.140)	<0.001	1.075 (1.032–1.120)	0.001
Female (no = 0, yes = 1)	2.509 (1.177–5.349)	0.017	Unentered	
Dialysis vintage (months)	0.998 (0.989–1.008)	0.742	Unentered	
With diabetes mellitus (no = 0, yes = 1)	1.878 (0.948–3.72)	0.071	Unentered	
With CVD history (no = 0, yes = 1)	3.176 (1.577–6.396)	0.001	Unentered	
Education (years)	0.725 (0.639–0.823)	<0.001	0.758 (0.650–0.883)	<0.001
DBP (mmHg)	0.972(0.943–1.002)	0.063	Unentered	
Serum albumin (g/L)	0.869 (0.778–0.970)	0.012	Unentered	
NLR	1.214 (0.987–1.493)	0.066	Unentered	
NPAR	11.634 (2.661–50.868)	0.001	9.952 (1.587–62.390)	0.014
Serum potassium (mmol/L)	0.547 (0.306–0.979)	0.042	Unentered	
Serum creatinine (umol/L)	0.997 (0.996–0.998)	<0.001	Unentered	
Serum phosphorus (mmol/L)	0.238 (0.100–0.569)	0.001	0.320 (0.111–0.922)	0.035

PD, peritoneal dialysis; CVD, cardiovascular disease; DBP, diastolic blood pressure; NLR, neutrophil-to-lymphocyte ratio; NPAR, neutrophil percentage-to-albumin ratio.

### 3.4 Evaluation of NPAR’S predictive ability for CI in PD patients

**Figure 2:**
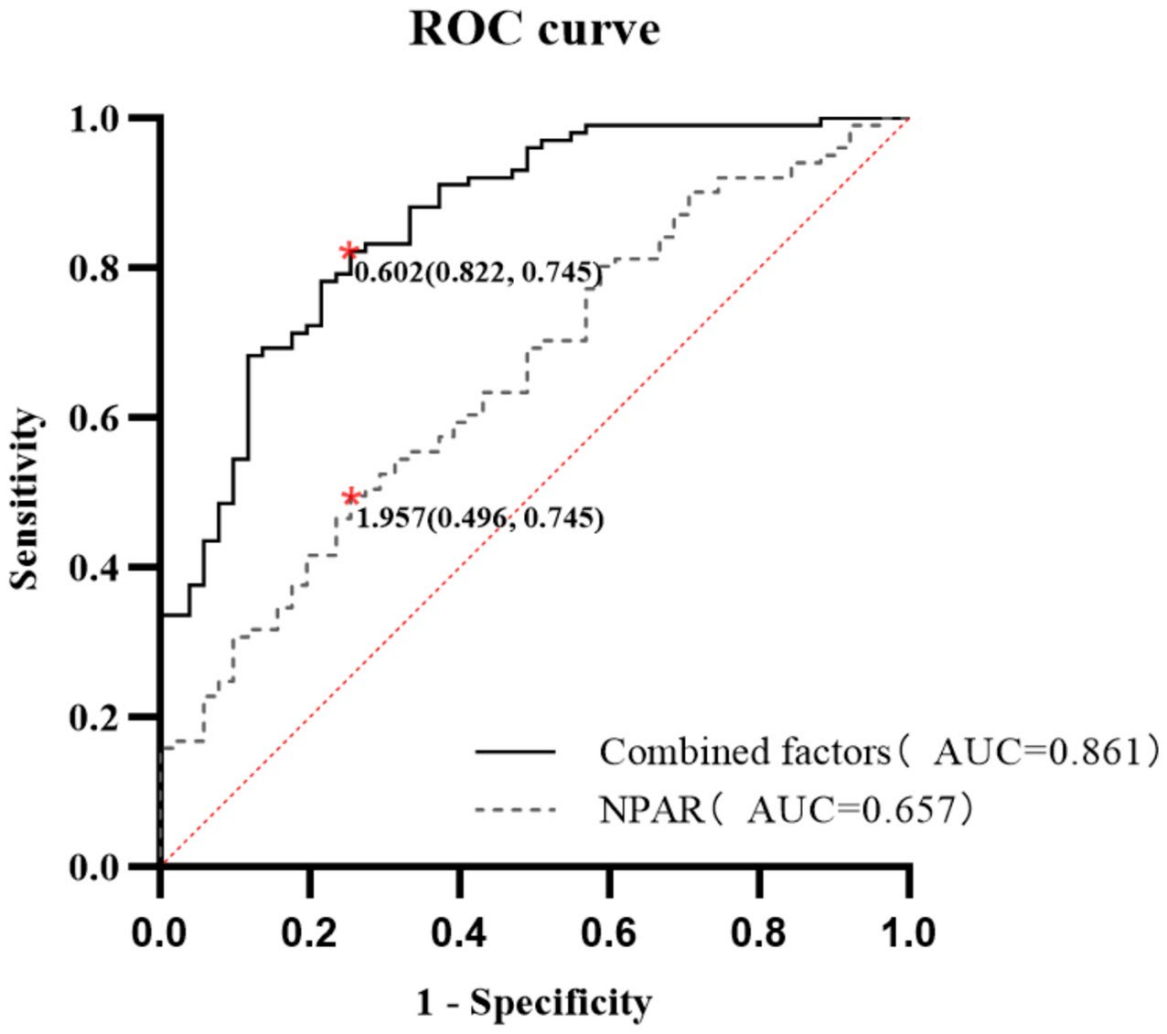
ROC curves of individual and combined predictors for cognitive impairment in PD patients. ROC curves were used to assess the predictive value of various factors for cognitive impairment in patients undergoing peritoneal dialysis. NPAR alone yielded an AUC of 0.657 (*p* < 0.01), with a sensitivity of 49.5% and a specificity of 74.5%. When age, education, NPAR, and serum phosphorus levels were combined, the AUC increased to 0.861, with a sensitivity of 88.2% and specificity of 72.5% (*p* < 0.01). ROC, Receiver operating characteristic; AUC, area under the curve; NPAR, neutrophil percentage-to-albumin ratio.

**Figure 3:**
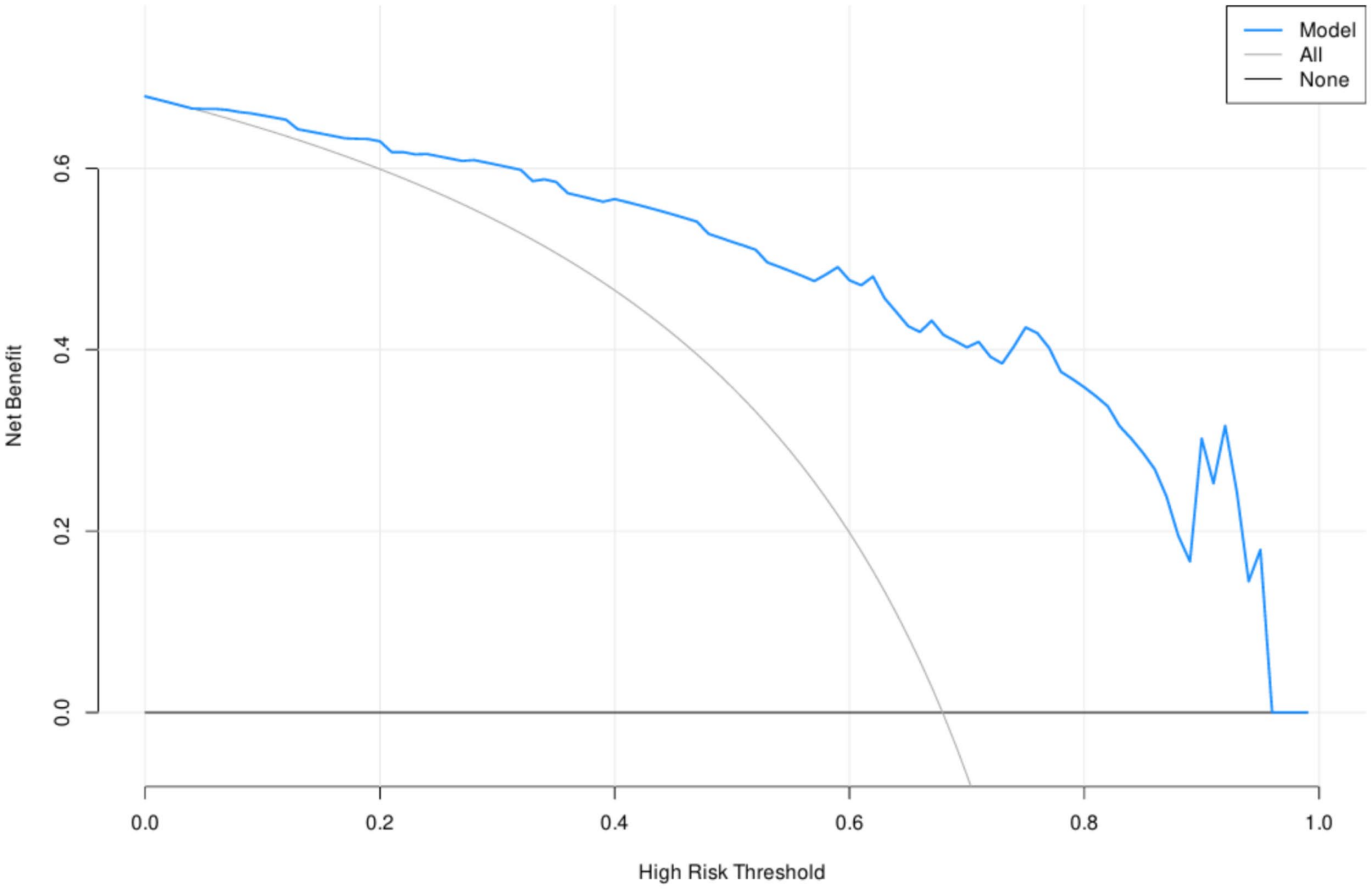
Decision curve analysis of predictors for cognitive impairment in PD patients.

The ROC analysis demonstrated that NPAR possessed moderate predictive capability for CI in PD patients, with an AUC of 0.657 (*p* = 0.002), a sensitivity of 49.6%, and a specificity of 74.5%. When combined with age, education, NPAR, and serum phosphorus, the predictive model’s AUC improved significantly to 0.861, exhibiting a sensitivity of 88.2% and a specificity of 74.5% (*p* < 0.001) (Table 3 and Figure 2). The DCA analysis demonstrated that the net benefit rate of the predictive model was higher than that of the simple model (Figure 3).

**Table 3:** Predictive performance of factors for NPAR in PD patients.

Factor	AUC	*p*-value	Sensitivity	Specificity	95%CI	
					Lower	Upper
Age (years)	0.781	<0.001	0.881	0.549	0.700	0.861
Education (years)	0.748	<0.001	0.802	0.529	0.170	0.334
NPAR	0.657	0.002	0.496	0.745	0.567	0.746
Serum phosphorus (mmol/L)	0.642	0.004	0.851	0.373	0.263	0.452
Combined factors	0.861	<0.001	0.822	0.745	0.800	0.922

NPAR, neutrophil percentage-to-albumin ratio; PD, peritoneal dialysis.

**Figure 4:**
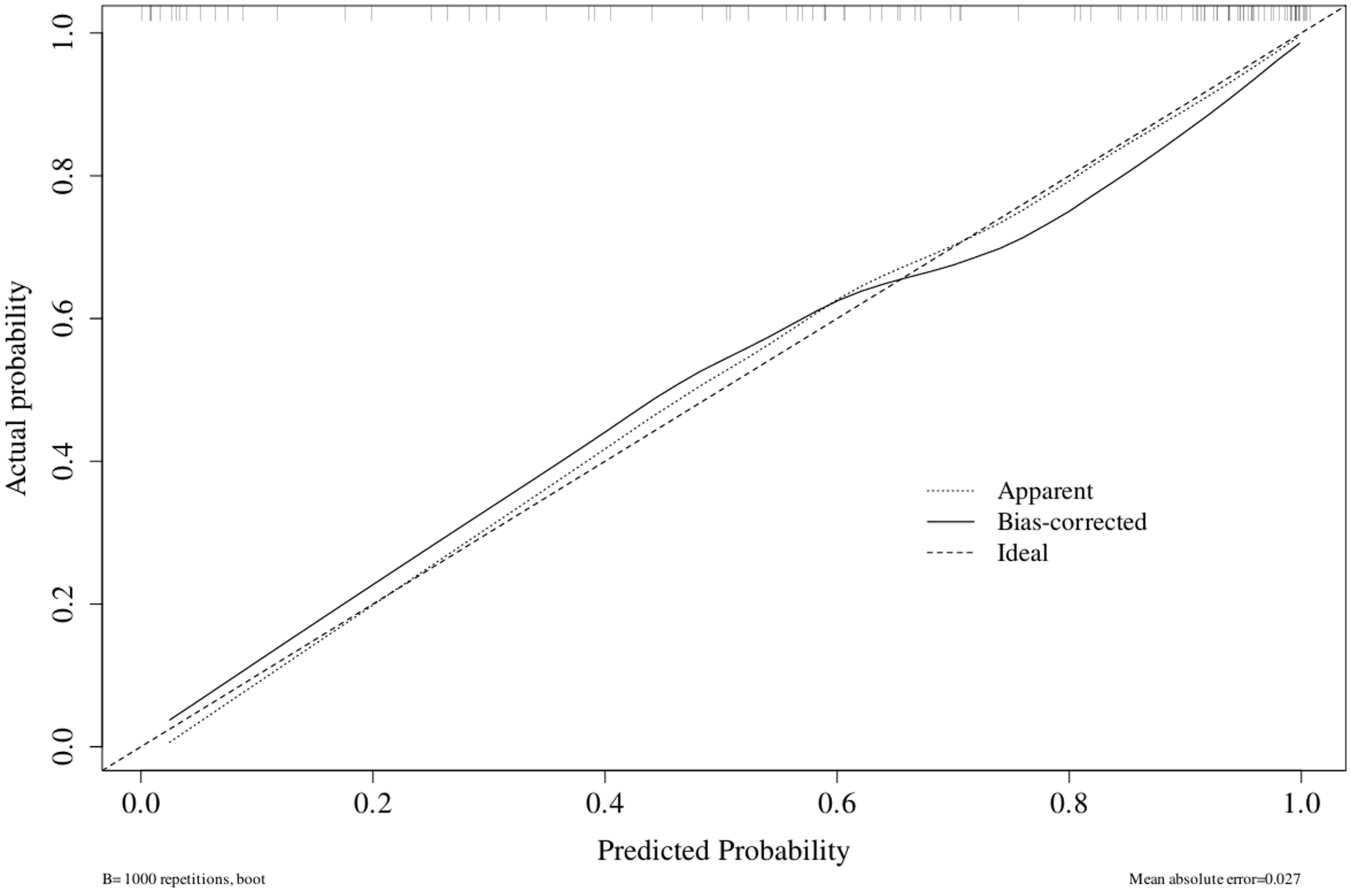
Calibration curve of the predictive model.

The model calibration assessment demonstrated that the bias-corrected calibration curve, derived from 1,000 bootstrap resamples, closely aligned with the ideal calibration line, indicating a strong agreement between predicted and observed outcome probabilities. The mean absolute calibration error across the bootstrap samples was 0.027. Furthermore, the Hosmer–Lemeshow goodness-of-fit test resulted in a *χ*^2^ statistic of 3.301 (df = 8, *p* = 0.914), indicating no significant deviation between the model predictions and actual observations (Figure 4).

## 4 Discussion

Our study demonstrated a significant link between the NPAR and the development of cognitive impairment in PD patients, independent of clinical characteristics, nutritional indicators, and inflammatory markers. To the best of our knowledge, this study is the first to demonstrate that the NPAR could serve as a reliable indicator of CI in patients undergoing peritoneal dialysis, thereby contributing valuable information for understanding the link between systemic inflammation and CI in this population.

CI is a common complication in patients undergoing PD. Various screening tools can be used for determining CI, and different methods yield varying prevalence rates. A meta-analysis showed that the highest prevalence was observed when using the Repeatable Battery for the Assessment of Neuropsychological Status (RBANS) [74.5% (95%CI:60.9–84.6%)], followed by the MoCA [64.6% (95%CI: 52.4–75.2%)], Addenbrooke’s Cognitive Examination (ACE) [64.6% (95% CI:52.4–75.2%)], Mental State Examination (MSE) [20.2% (95%CI:16.1–25.1%)], and Trail-Making Test (TMT) [18.8% (95%CI:15.8–22.3%)] (3). Another study found that the MoCA scale had the highest overall predictive ability for CI in dialysis patients (14). Therefore, our study used MoCA tests as a preliminary assessment tool for assessing CI in PD patients, resulting in a prevalence of 66.45%, which is consistent with the prevalence rates reported in previous meta-analyses using the MoCA scale (3).

The pathogenesis of CI in dialysis patients is not yet fully understood, but it mainly includes uremic toxins, vascular lesions, inflammation, and malnutrition. Recently, the association between inflammation and CI has received increased attention. Neutrophils are a core indicator of systemic chronic inflammation and play a key role in CI pathogenesis. Previous studies reported that an increase in peripheral neutrophils is associated with neuroinflammation, cerebral microvascular obstruction, and amplified oxidative stress in neurodegenerative diseases (15–17). Additionally, targeted inhibition of neutrophil-mediated pathological processes has been shown to improve cognitive deficits (17, 18). Albumin, the primary plasma protein, integrates nutritional status and anti-inflammatory capacity, exerting protective effects on cognitive function by reducing neuronal injury and tau hyperphosphorylation. A decrease in albumin levels is closely linked to an increased risk of CI in elderly and uremic populations (19, 20).

Therefore, neutrophils and albumin have a notable impact on the initiation and advancement of CI. The NPAR, derived from the percentage of neutrophils and albumin levels, encompasses two important immune pathways within the body, serving as a recent indicator of the systemic inflammatory response and overall immune status. An elevated NPAR level indicates higher neutrophil percentages and lower albumin levels, suggesting that the NPAR can potentially serve as a valuable predictor of CI. We first confirmed that a higher NPAR is independently associated with CI in PD patients. As a potential associated biomarker for CI, the NPAR offers a distinct clinical advantage in that it can be easily and rapidly derived from routine blood tests that are routinely performed during the follow-up of PD patients, without requiring additional specialized assessments or medical resources.

Previous studies have shown that many factors affect the occurrence of CI in PD patients, such as age, sex, education, CVD history, diabetes mellitus, hyponatremia, and vascular calcification, which is consistent with our comparative results between the CI and NCI groups (21–23). Another finding of our study was that patients in the CI group had lower serum phosphorus levels, which emerged as a protective factor for CI in the multivariate regression analysis. We speculated that this result may be partially explained by confounding factors such as age. A previous study found that serum phosphate levels gradually decreased with age in PD patients (24). In our study, the proportion of elderly patients (≥ 65 years) was higher in the CI group compared to the NCI group (50.5% vs. 13.7%, *p* < 0.001), and there was an inverse correlation between serum phosphorus levels and age in PD patients. Therefore, we speculated that lower serum phosphorus levels in the CI group may be related to age. In addition, there were more patients with hypophosphatemia in the CI group, while hypophosphatemia always indicated malnutrition and was associated with increased mortality in patients undergoing dialysis, which may explain the relationship between phosphorus levels and CI (25, 26). However, this finding is exploratory and requires further validation in larger cohorts.

In our study, we evaluated the predictive abilities of the NPAR in comparison to other markers such as the NLR and CAR. We found that only the NPAR displayed strong independent predictive power for CI in PD patients, while the NLR and CAR showed no significant differences between the CI and NCI groups. We speculated that this discrepancy may be attributed to the unique compositional advantages of the NPAR. Compared to the NLR, the NPAR used neutrophil percentage rather than the absolute count, which minimized interference from acute factors (such as transient infections and dialysis-related hemodynamic changes) and better reflected the chronic low-grade inflammation inherent to PD. In addition, albumin integrated nutritional status and anti-inflammatory capacity, which are key factors in malnutrition-inflammation complex syndrome associated with PD (9). However, the CAR relied on acute-phase CRP, while the NLR may be confounded by lymphocyte instability due to comorbidities or immunosuppression. Thus, the NPAR’s combination of a stable chronic inflammation indicator and a dual nutrition-inflammation marker makes it more relevant for predicting CI in PD patients. Furthermore, incorporating factors such as age, education level, and serum phosphate levels into the model substantially improved its predictive accuracy, achieving an AUC of 0.861. These findings may help in the early identification of CI in PD patients and may further contribute to improving and delaying the progression of CI.

Our study has some limitations. First, this study was a single-center observational study with a relatively small sample size of 152 PD patients, which inevitably restricts the generalizability of our results to the broader PD population. Second, due to the cross-sectional nature of this study, it was not possible to ascertain the cause-and-effect link between the NPAR and cognitive function in PD patients. Third, it failed to comprehensively exclude other influencing factors through imaging examinations. Importantly, we did not collect data on depression and sleep disorders, which are two highly prevalent conditions in dialysis patients and are strongly associated with cognitive impairment. Their absence as candidate variables in the multivariate model may introduce residual confounding, which could affect the robustness of our findings. In addition, although the combined predictive model of age, education, NPAR, and serum phosphorus level yielded a relatively high AUC value, it should be noted that this model has a potential risk of overfitting due to the relatively small sample size of the current single-center study. The optimal cutoff values and predictive performance of this model need to be further validated in an external independent cohort to confirm its clinical applicability. Further studies are needed to confirm the potential mechanisms by which the NPAR affects CI in PD patients.

## 5 Conclusion

An increased NPAR is associated with the development of cognitive impairment in PD patients and may serve as a potential biomarker for its detection. However, further longitudinal studies are needed to verify whether the NPAR can predict the onset of CI and inform early interventions.

## Data Availability

The original contributions presented in the study are included in the article/supplementary material, further inquiries can be directed to the corresponding author.
